# Efficacy of Tildrakizumab for the Treatment of Difficult-to-Treat Areas: Scalp, Nail, Palmoplantar and Genital Psoriasis

**DOI:** 10.3390/jcm11092631

**Published:** 2022-05-07

**Authors:** Marco Galluzzo, Marina Talamonti, Arnaldo Cioni, Virginia Maffei, Ruslana Gaeta Shumak, Lorenzo Tofani, Luca Bianchi, Elena Campione

**Affiliations:** 1 Department of Systems Medicine, University of Rome “Tor Vergata”, 00133 Rome, Italy; talamonti.marina@gmail.com (M.T.); arnaldo.cioni@hotmail.com (A.C.); virginiamaffei1@gmail.com (V.M.); ruslanagaetashumak@gmail.com (R.G.S.); lorenzotofani1993@gmail.com (L.T.); luca.bianchi@uniroma2.it (L.B.); campioneelena@hotmail.com (E.C.); 2 Dermatology Unit, Fondazione Policlinico “Tor Vergata”, 00133 Rome, Italy

**Keywords:** psoriasis, tildrakizumab, PASI, NAPSI, PSSI, sPGA-G, ppPASI, real-life, difficult locations

## Abstract

Tildrakizumab, an IL-23 inhibitor, is effective and safe for the improvement of moderate-to-severe chronic plaque psoriasis. However, little evidence is available on the use of this biologic in psoriasis in difficult-to-treat locations. In this retrospective analysis, we treated patients with 100 mg tildrakizumab at Day 0, after 4 weeks and every 12 weeks thereafter. Disease severity and treatment response was assessed by the Psoriasis Area and Severity Index (PASI), the static Physician’s Global Assessment of Genitalia (sPGA-G), the Psoriasis Scalp Severity Index (PSSI), Nail Psoriasis Severity Index (NAPSI) and the Palmoplantar Psoriasis Area and Severity Index (ppPASI) at baseline and after 4, 12 and 28 weeks. We followed 18 patients (mean age 49.1 ± 12.7 years, 61.1% male) with psoriasis localized to the genital region (N = 7), scalp (N = 6), nails (N = 5) and palmar/plantar areas (N = 7). PASI score decreased from 11.5 at baseline to 3.1 and 2.4 at 12 and 28 weeks. Tildrakizumab treatment decreased sPGA-G (3.3 to 0.2), PSSI (36.2 to 2.7), NAPSI (48.4 to 15.7) and ppPASI (5.3 to 0) from baseline to 28 weeks, respectively. Data from this real-life retrospective analysis shows that tildrakizumab is an effective option for the management of psoriasis in difficult-to-treat areas.

## 1. Introduction

Plaque psoriasis is a chronic, inflammatory immune skin disease affecting 2–3% of the population worldwide [1] and is associated with detrimental physical effects, disability, reduced psychological wellbeing and impaired quality of life (QoL) [2,3].

Psoriasis often affects areas that are difficult to treat, such as the genital areas (14–43%), scalp (43–65%), face (30–49%), nails (23–60%), as well as hands and/or feet (12–16%) [4,5,6,7,8,9,10,11].

Psoriasis affecting genital areas may be associated with considerable morbidity, discomfort and embarrassment and may significantly impair the QoL and psychosexual wellbeing of patients [12]. Traditionally, low-to-mild-potency topical corticosteroids, topical calcineurin inhibitors and vitamin D analogues are recommended as first-line treatment for genital psoriasis. In cases of recalcitrant or severe genital psoriasis, biologic and other systemic therapies are recommended [13].

Scalp psoriasis is characterized by demarcated erythematosquamous chronic plaques that often advance beyond the hairline into the face and retroauricolar area. Due to its visibility, scaling of the scalp strongly impacts QoL, frequently causing embarrassment and psychosocial handicap. Similar to genital psoriasis, first-line treatment for scalp psoriasis includes topical therapies such as corticosteroids, vitamin D3 analogues and a combination of products with systemic therapy used in severe cases [14].

Patients with psoriatic arthritis frequently present with nail psoriasis (>80% of patients), and this disease is often resistant to topical treatment, which has a major impact on the patient’s QoL [15,16]. While conventional systemic treatments such as methotrexate, cyclosporine and acitretin are shown to be effective, the treatment of nail psoriasis still remains a challenge [17].

Psoriatic lesions affecting the palms and soles (palmoplantar) strongly impact patient QoL while also compromising their ability to work and leading to social embarrassment/stigmatism. Initial treatment is based on the use of high-potency topical steroids and phototherapy, and in cases where systemic therapy is required, the most used traditional agents are methotrexate and acitretin [18].

Since patients with psoriasis in difficult-to-treat locations often receive inadequate treatment, the burden of the disease is often worse than patients not affected by these types of lesions. In general, topical treatments are not usually effective necessitating alternative treatment strategies.

Biological therapies such as tumor necrosis factor (TNF), interleukin (IL) 12/23 and IL-17 inhibitors have revolutionized the management of moderate-to-severe psoriasis allowing many patients to attain clear skin [19]. Although evidence is limited, several biologics are shown to be able to effectively treat psoriasis in some difficult-to-treat areas [20,21,22,23,24,25]. Tildrakizumab is a humanized IgG1 κ monoclonal antibody targeting the p19 subunit of IL-23 and is currently approved for the treatment of moderate-to-severe plaque psoriasis in patients eligible for systemic therapy [26,27,28]. Although there is accumulating evidence of the effectiveness of tildrakizumab in randomized controlled trials (RCTs) [29,30] and more recently in real-life studies [31,32,33,34], little evidence is currently available on the use of this biologic in patients burdened with psoriasis in difficult-to-treat areas [35]. In this retrospective analysis, we evaluated the efficacy of tildrakizumab in a small cohort of patients with psoriasis in difficult-to-treat locations.

## 2. Methods

### 2.1. Patients and Study Design

A total of 38 patients were treated with tildrakizumab at our Dermatology Unit, “Tor Vergata” University of Rome, up to 27 April 2021. Following a review of data from medical records, 18 patients were identified as having involvement in at least one difficult-to-treat area. We conducted a 28-week retrospective study designed to evaluate the effectiveness of tildrakizumab in difficult sites. Tildrakizumab was administered at the standard dosing regimen (induction phase: 100 mg subcutaneously at weeks 0 and 4, and a maintenance dose every 12 weeks thereafter) to patients with moderate-to-severe psoriasis (Psoriasis Area Severity Index (PASI) > 10) who failed to respond or had contraindications or side effects to at least one conventional treatment, including systemic therapy (i.e., methotrexate, cyclosporine, or acitretin) or phototherapy according to Italian guidelines for the prescription of biologic therapy [36]. Patients with a baseline PASI < 10 who presented the involvement of difficult-to-treat areas such as the face, scalp, hands or genital areas were also considered eligible for treatment with tildrakizumab. All patients gave written informed consent for their participation prior to enrolment. This study complies with the ethical standards laid down in the 1975 Declaration of Helsinki.

### 2.2. Outcome Measures

Safety and efficacy were assessed at weeks 0, 4, 12 and 28 using the psoriasis area severity index (PASI), static Physician’s Global Assessment of Genitalia (sPGA-G) [37], Psoriasis Scalp Severity Index (PSSI) [38], Nail Area Psoriasis Severity Index (NAPSI) [39] and the Palmoplantar Psoriasis Area and Severity Index (ppPASI) [40].

### 2.3. Safety

The safety and tolerability (including the presence of any adverse events, AEs) of tildrakizumab were evaluated over the duration of the study. Clinical laboratory tests and control of vital signs were also assessed. Laboratory examinations (complete blood count, alanine aminotransferase, aspartate aminotransferase, gamma-glutamyltransferase, creatinine, high-density lipoprotein and low-density lipoprotein cholesterol, triglycerides, urea nitrogen, glucose, uric acid, C-reactive protein, lactate dehydrogenase and creatine phosphokinase) were performed at baseline and performed again after 1 year. If there are no alterations in blood tests at baseline, no further blood tests are required before 1 year of treatment with this new class of drugs (anti-IL23p19) in clinical practice. Quantiferon-TB gold tests and serology for hepatitis B virus, hepatitis C virus and human immunodeficiency virus were performed at baseline and at week 52.

Safety and tolerability were investigated by examining AEs, including mild and serious AEs, reported by patients and physicians during physical examinations at 4, 12 and 28 weeks. 

### 2.4. Statistical Analysis

Data are presented as a mean ± standard deviation for continuous variables and as a number and percentage for categorical variables. No formal statistical analysis was performed due to the small sample size involved.

## 3. Results

### 3.1. Patient Demographics and Clinical Characteristics

In this real-life retrospective analysis, 18 patients with moderate-to-severe plaque psoriasis involving difficult-to-treat areas were treated with tildrakizumab and followed over a period of 28 weeks. The baseline characteristics of patients are presented in Table 1. The majority of patients were male (61.1%) aged 49.1 ± 12.7 years with a long history of psoriasis (mean disease duration of 14.3 ± 11.9 years). Most patients were naïve to biologic treatment (N = 14; 77.8%). Hypertension and obesity were among the most frequent comorbidities (33.3% for both).

### 3.2. Treatment Response

**Figure 1:**
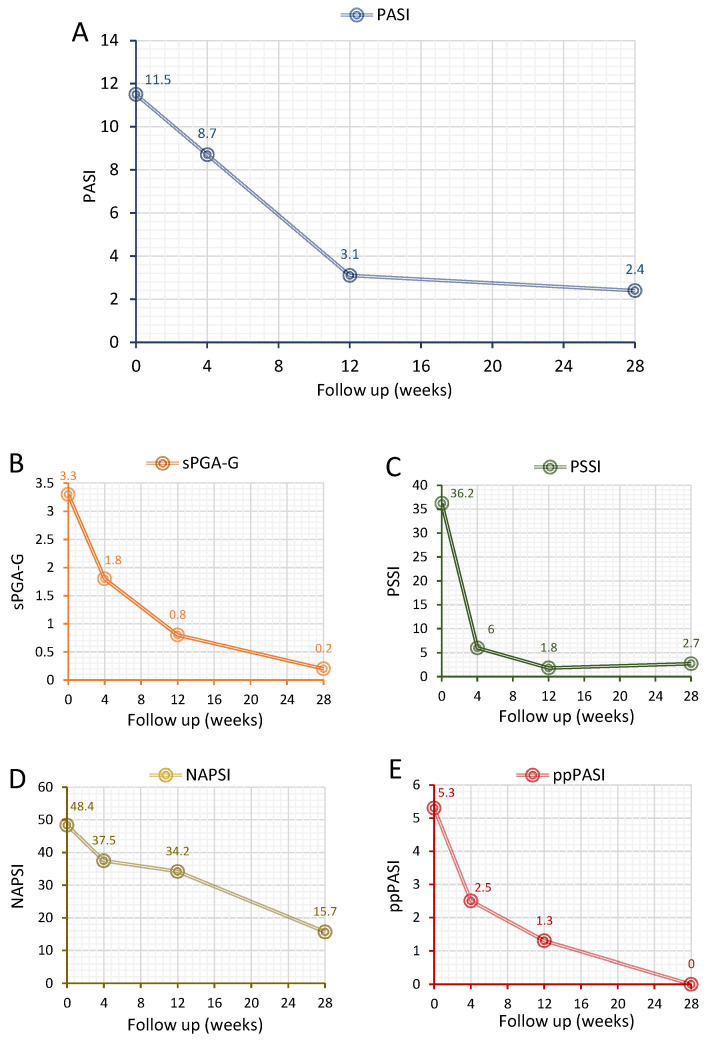
Effect of tildrakizumab in psoriatic patients on disease activity measures. (**A**) PASI response, (**B**) sPGA-G response, (**C**) PSSI response, (**D**) NAPSI response and (**E**) ppPASI response. Data are presented as mean scores based on 18 patients treated with tildrakizumab over 28 weeks. PASI = psoriasis area severity index; sPGA-G = static Physician’s Global Assessment of Genitalia; PSSI = Psoriasis Scalp Severity Index; NAPSI = Nail Area Psoriasis Severity Index and ppPASI = Palmoplantar Psoriasis Area and Severity Index.

In all 18 patients, treatment with tildrakizumab decreased a range of disease activity measures (Figure 1). The mean PASI score decreased from 11.5 at baseline to 3.1 at 12 weeks (equivalent to a 73% reduction) and 2.4 at 28 weeks (79.1% decrease) (Figure 1A). The sPGA-G score decreased from 3.3 at baseline to 0.8 at 12 weeks (decreased by 93.9%) and to 0.2 (by 93.9%) at week 28 (Figure 1B). The PSSI score for scalp involvement decreased from 36.2 to 6 by 4 weeks (83.4% decrease) and then to 1.8 and 2.7 at 12 and 28 weeks (95% and 92.5% decrease, respectively) (Figure 1C). The NAPSI score for nail involvement decreased to a lesser extent compared to other outcome measures but was decreased by 29.3% at week 12 (from 48.4 to 34.2) and by 67.6% at week 28 (from 48.4 to 15.7) (Figure 1D). The ppPASI score for palmoplantar involvement decreased by 75.5% at week 12, with complete clearance achieved by week 28 (Figure 1E).

### 3.3. Representative Cases Showing Improvement in Difficult-to-Treat Areas after Tildrakizumab

We describe three cases in detail that best represent the 18 patients treated with tildrakizumab in this retrospective analysis. 

#### 3.3.1. Case #1

**Figure 2:**
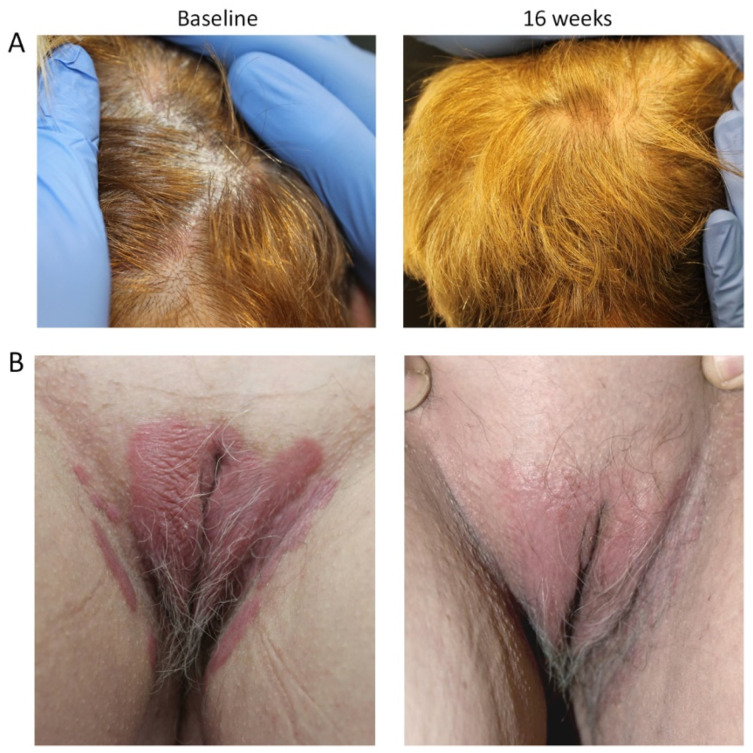
Representative images of outcome in Case #1; female patient aged 63 years, with severe scalp (**A**) and genital (**B**) psoriasis. After 16 weeks of tildrakizumab treatment, PASI decreased to 0.6, sPGA-G to 1, NAPSI to 0 and PSSI to 0.

Case #1 was a female patient aged 63 years, BMI of 23 kg/M2 with the onset of chronic plaque psoriasis at 23 years of age (40 years disease duration), cigarette smoker (20 cigarettes/day) with hypertension (Figure 2). In July 2020, she presented with severe scalp (Figure 2A) and genital (Figure 2B) psoriasis as well as nail involvement. She was previously treated with methotrexate (10 mg subcutaneously; s.c.) from February to May 2020, and this was suspended due to elevated transaminase levels (2-fold higher than the normal range). At the baseline visit (July 2020), the patient had a PASI score of 6, sPGA-G of 4, NAPSI of 6 and PSSI of 21. After 16 weeks (November 2020), her PASI decreased to 0.6, sPGA-G to 1, NAPSI to 0 and PSSI to 0. The patient has currently reached her 76th week of treatment and is disease-free.

#### 3.3.2. Case #2

**Figure 3:**
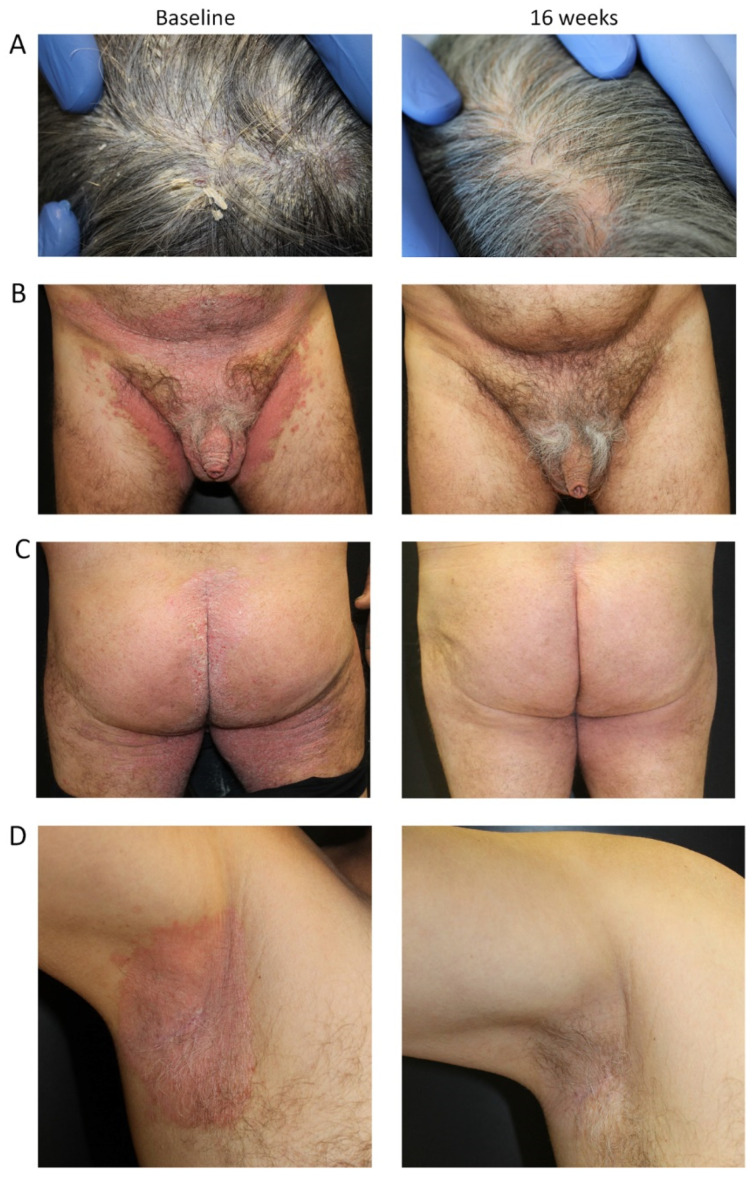
Representative images of outcome in Case #2; male patient with psoriatic lesions of the scalp (**A**), genital lesions (**B**) and involvement of intertriginous areas (gluteal; (**C**) and armpit; (**D**)). After 16 weeks of treatment with tildrakizumab, all outcome measures (PASI, sPGA-G and PSSI) decreased to 0.

The second patient (Case #2) was a male aged 62 years, with a BMI of 30.5 kg/M^2^ and the onset of chronic plaque psoriasis at 30 years of age (32 years disease duration), cigarette smoker (10 cigarettes/day for the past 30 years) and QuantiFERON-TB Gold + (Figure 3). He was previously treated with methotrexate (10 mg, s.c.) cycles from July 2017 to March 2019 with good response but experienced a rapid return of the disease upon suspension. Secukinumab (300 mg) was initiated in April 2019 (PASI score of 20) until July 2020 (PASI score of 2) but suspended at the request of the patient due to the constant presence of psoriatic lesions in intertriginous areas (armpits and groin areas) and episodes of recurrent oral candidiasis frequently non-responsive to topical treatment or probiotic therapies. In November 2020, following the marked deterioration of his clinical condition, he was started on tildrakizumab (100 mg) to treat severe lesions of the scalp (Figure 3A), genital area (Figure 3B) and intertriginous areas (Figure 3C,D). At the baseline visit (November 2020), the patient had a PASI score of 12, sPGA-G of 5 and PSSI of 60. After 16 weeks (February 2021), all outcome measures (PASI, sPGA-G and PSSI) decreased to 0. The patient has now been treated with tildrakizumab for 1 year and is completely free of disease, with no relapse of disease during the course of treatment or development of recurrent candida infection (as occurred during treatment with secukinumab).

#### 3.3.3. Case #3

**Figure 4:**
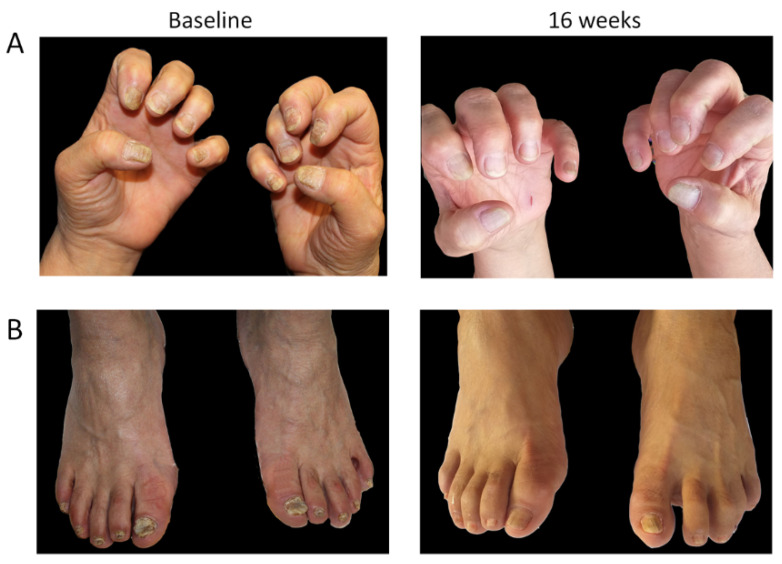
Representative images of outcome in Case #3; a female patient with severe hand (**A**) and foot (**B**) nail involvement. After 16 weeks of tildrakizumab treatment, both PASI score and NAPSI index decreased from 10 to 0 and from 160 to 86, respectively.

The third case (Case #3) described is a female patient aged 62 years, with a BMI of 30.5 kg/M^2^ and the onset of chronic plaque psoriasis with severe nail involvement (both hands and feet) at 46 years of age (16 years of disease duration), cigarette smoker (20 cigarettes/day), with hypertension, hypothyroidism and glaucoma (Figure 4). She was previously treated with topical corticosteroids and vitamin D-based ointments. The patient initiated tildrakizumab treatment in January 2021 (PASI of 10 and NAPSI of 160), and after 16 weeks (May 2021), the PASI score decreased to 0 and NAPSI decreased to 86. The patient has now reached 1 year of treatment, maintaining a PASI index of 0. During treatment, there was a further improvement in the NAPSI index, with the last value recorded in week 48 of 42.

### 3.4. Safety

Tildrakizumab was generally well tolerated without evidence of cumulative toxicity or organ toxicity. No patients dropped out of the treatment due to AEs, which were not reported.

## 4. Discussion

This real-life study of 18 psoriasis patients showed that 100 mg of tildrakizumab was effective over 28 weeks for the treatment of chronic plaque psoriasis in difficult-to-treat areas, including the genitals, scalp, nails and palmoplantar areas.

Our findings showed a marked improvement in the outcome measures sPGA-G, PSSI, NAPSI and ppPASI without the presence of adverse events associated with tildrakizumab treatment.

While several studies examined the efficacy of biologics such as tildrakizumab in specific sites that are difficult to treat, few studies examined the effect of biologics to treat multiple locations that are difficult to treat in a real-life setting.

In patients that are burdened with genital psoriasis, few studies evaluated the effects of systemic biologics in these patients.

Although evidence from trials [29,30] and real-life studies [31,32,33] demonstrated that tildrakizumab has a favorable efficacy and safety profile for the treatment of moderate-to-severe psoriasis that persists up to at least 5 years [41], real-life experience on the use of this biologic for genital psoriasis is limited.

Ixekizumab was the first biologic demonstrating efficacy and safety in a formal clinical trial on genital psoriasis [42], while the ongoing GULLIVER study is evaluating the efficacy of guselkumab in moderate facial and genital psoriasis (NCT04439526) [43]. Our real-life experience is the first to provide real-life data on the efficacy of tildrakizumab in genital psoriasis. After 28 weeks, sPGA-G score decreased by 93.9%, leading to almost complete remission of genital psoriasis without any adverse events reported. 

In addition to an improvement in genital psoriasis, we also observed a pronounced improvement in patients with scalp involvement. The effectiveness of other biologics for the treatment of scalp psoriasis was previously evaluated. In a 24-week RCT, Bagel et al. showed an improvement in PSSI of 86.8% in patients treated with etanercept vs. 20.4% in the placebo group [44]. Infliximab was also shown to be effective for the treatment of scalp psoriasis, as documented by results from three controlled trials describing the efficacy of Infliximab in the scalp region [25]. Furthermore, a post hoc analysis of the BELIEVE RCT showed that after 16 weeks, adalimumab afforded a reduction in PSSI score by 77.2% [22].

Ixekizumab at doses of 75 and 150 mg appeared to show comparable results to anti-TNF-alpha with complete resolution of scalp psoriasis plaques in almost 80% of patients [45]. In a pooled analysis of four phase 3 studies, secukinumab showed strong and sustained efficacy for the head and neck over 52 weeks, with a head and neck PASI 90/100 subscore achieved by 76.0%/68.7% of patients receiving 300 mg secukinumab, respectively, and by 61.4%/53.1% of patients receiving 150 mg secukinumab, respectively [46]. In a large, pooled psoriasis dataset from the VOYAGE 1 and 2 studies, guselkumab was superior to adalimumab for the treatment of psoriasis of the scalp with near-complete or complete clearance of the scalp (85.0% vs. 68.5% for scalp-specific Investigator Global Assessment; ss-IGA 0/1) [24]. A phase 2 trial comparing risankizumab and ustekinumab in patients with moderate-to-severe plaque psoriasis observed a reduction in PSSI score at week 12 of 90% in the 90 mg risankizumab group and 94% in the 180 mg risankizumab group that was sustained over time, compared with an 82% reduction in the ustekinumab group that was not maintained over time [47]. To our knowledge, no published RCTs have specifically investigated the effect of ustekinumab on scalp psoriasis. A post hoc analysis of reSURFACE 1 evaluated the effect of 100 mg tildrakizumab or placebo on scalp, head and neck psoriasis over 28 weeks in adult patients with-moderate-to-severe chronic plaque psoriasis [48]. Over 28 weeks, the Psoriasis Area and Severity Index head component scores (PASIh) decreased rapidly for all 309 patients and 166 (21.5%) patients had PASIh score of 0 by 28 weeks [48]. A phase 3b study of 100 mg tildrakizumab for scalp psoriasis is currently ongoing [49]. In our experience, the PSSI score for scalp involvement decreased by 83.4% at week 4, by 95% at week 12 and by 92.5% at week 28. Although these findings are preliminary and the sample size is small, our experience in the real-life setting confirms the efficacy and safety of tildrakizumab in the treatment of scalp psoriasis as demonstrated by clinical trials.

To date, no biologic agents have a specific indication for nail psoriasis. The first RCT to have nail involvement as its primary endpoint evaluated the efficacy of adalimumab and demonstrated its efficacy with significant improvement in moderate-to-severe nail psoriasis vs. placebo [23]. Adalimumab was also evaluated vs. guselkumab in the VOYAGE studies and was not found to be inferior [24]. IL-17 antagonists were evaluated in two RCTs: The first, the TRANSFIGURE RCT, demonstrated a strong and long-lasting efficacy of secukinumab up to 2.5 years with an improvement in QoL and good safety profile [20]; the second, the IXORA-S Head-to-Head Study compared the efficacy of ixekizumab and ustekinumab demonstrating the superiority of ixekizumab in achieving complete clearance [21]. A recent systematic review conducted also concluded that tofacitinib and ixekizumab are the most effective agents to treat nail psoriasis [50]. We recently documented the long-term efficacy of ustekinumab for the treatment of crumbly nail psoriasis in a 69-year-old man [51]. The patient had severe onychodystrophy, onycholysis and pain in the hands (NAPSI score of 69). Ustekinumab, in combination with methotrexate, improved nail psoriasis (NAPSI of 0 at 104 weeks) and pain caused by synovitis over a period of 4 years.

In terms of evidence to date evaluating the use of tildrakizumab, there are no RCTs evaluating its efficacy in nail disease, although some real-life experiences suggest that tildrakizumab may be a valid treatment option. Ismail et al. reported the case of a man with psoriatic nail dystrophy and psoriatic arthritis with an excellent clinical response both on nail disease and arthritis [52]. Simpson and colleagues reported two cases which showed the strong efficacy of tildrakizumab in treating nails, demonstrating a reduction of mNAPSI of more than 75% at 12 months [35]. 

In our experience, 5 out of 19 patients had nail involvement with a mean baseline NAPSI score of 48.4. At week 28, the mean NAPSI score decreased to 15.7, corroborating results from these other real-life experiences and confirming that tildrakizumab could be an effective therapeutic choice for the treatment of this difficult site.

Biological therapy for palmoplantar psoriasis still remains without a precise indication, and some trials demonstrated the efficacy of anti-TNF alpha agents, IL-17 and Il-23 inhibitors, but there is still no common consensus on which agent is the most effective. In a systematic review conducted in 2017 from multiple large RCTs, adalimumab, guselkumab, ixekizumab, secukinumab and ustekinumab all showed >80% efficacy for the treatment of hyperkeratotic palmoplantar psoriasis compared to placebo. Tildrakizumab and risankizumab are yet to be evaluated for palmoplantar involvement.

Few trials specifically evaluated the efficacy of biologics in patients with palmoplantar psoriasis. In the GESTURE trial, 205 patients were randomized to 150 mg or 300 mg secukinumab or placebo [23]. The primary endpoint was palmoplantar Investigator’s Global Assessment (ppIGA) 0 or 1, corresponding to ‘clear’ or ‘almost clear’ palms and soles skin at 16 weeks. At 16 weeks, significantly more patients treated with 300 mg or 150 mg secukinumab had ‘clear’ or ‘almost clear’ palms and soles skin compared with placebo group patients (respectively, 33.3%, 22.1% and 1.5%, *p* < 0.001). ppPASI also improved with both doses of secukinumab vs. placebo (−54.5%, −35.3%, −4.0%, *p* < 0.001, respectively). These disease improvements had a positive impact on health-related QoL as measured by the Dermatology Life Quality Index (DLQI). The GESTURE study also evaluated the long-term (2.5-year) efficacy and safety and showed that the effects seen at 16 weeks were sustained, with 53% and 59% of patients reaching 2.5 years for the primary endpoint of mean ppIGA 0 or 1, respectively, for 300 mg and 150 mg secukinumab [24]. 

In our real-life study, we also showed that secukinumab is effective in the treatment of palmoplantar psoriasis [53]. Forty-three patients were treated with secukinumab. Previous treatments included topical and systemic therapies, and 50% of patients were previously treated with a biologic. Mean PASI was rapidly improved after secukinumab treatment, with a 78.2% decrease observed at 16 weeks. Mean ppPASI also substantially improved, but the change was more gradual, with reductions of 55% and 79.3% at 16 and 104 weeks, respectively. Approximately half of the patients achieved complete skin clearance at 40 weeks [53].

Our real-life experience is the first evidence documenting the efficacy of tildrakizumab in psoriasis involving this difficult-to-treat area. In our experience, seven patients had palmoplantar involvement with a mean baseline ppPASI of 5.3. After 28 weeks, the ppPASI decreased to 0, suggesting that tildrakizumab may also be a valid treatment option for palmoplantar psoriasis.

It is also important to highlight that in this retrospective analysis, 14 out of 18 patients (77.8%) were naive to biologics. Therefore, it cannot be ruled out that the rapid action observed and the excellent response are linked to the use of this drug as a first-line treatment. Furthermore, the issue of early treatment and treat-to-target should not be underestimated in patients who have nail involvement, which is recognized as the first red flag for the onset of psoriatic arthritis.

Indeed, several studies observed a positive association between interphalangeal, distal interphalangeal (DIP), proximal interphalangeal and metacarpophalangeal (MCP) joint involvement of the hands and psoriatic nail changes [54]. It is also recognized that nail changes stand for an enthesitis of the nail bed and matrix, forming a unique functional complex referred to as the synovial–enthesial complex [55].

Using MRI imaging in PsA patients, Tan and colleagues demonstrated the close relationship between the nail bed, insertion of the extensor tendon, the DIP joint and the distal phalanx. They also showed that the inflammation typically begins in the nail and moves proximally to the distal phalanx and later to the DIP joint. This supports the observation that skin disease often precedes joint disease in patients with PsA [56]. In addition, it is known that the nail is anchored to the bone via entheses, and cytokines such as IL-23, IL-17 and IL-22 affect these structures via the entheses.

Collectively, our real-life observations demonstrate the rapid and effective improvement of psoriasis in multiple difficult-to-treat locations. Further studies evaluating the persistence of this benefit in the long term as well as potential differences in patients provided with tildrakizumab as a first or second-line biologic are warranted.

## 5. Study Limitations and Strengths

The main limitations of this study are the small sample size (N = 18) and relatively short follow-up period of 28 weeks. The small number of patients precluded the possibility of performing formal statistical analysis, and although post hoc analysis of resurface-1 and -2 shows the long-term efficacy of tildrakizumab [57], further studies are needed to evaluate the long-term efficacy of tildrakizumab in these difficult-to-treat patients. In our small cohort, 14 patients (77.8%) were biologic naïve, and this may have improved the response observed by tildrakizumab. However, Bonifati and colleagues provided evidence in a small real-life cohort that in cases of primary or secondary failure to IL-17 inhibitors, class switching to IL-23 inhibitors can provide adequate response [58]. The strengths of this study were that it was undertaken in real-life clinical practice in patients burdened by psoriasis localized in multiple difficult-to-treat areas.

## 6. Conclusions

In our real-life experience over a period of 28 weeks, tildrakizumab was shown to be extremely effective for the treatment of moderate-to-severe chronic plaque psoriasis involving difficult-to-treat areas with a significant improvement of PASI, NAPSI, sPGA-G, PSSI and ppPASI. The safety profile was shown to be good in all patients, with no AEs being reported. Further long-term studies with a greater sample size are needed to verify these preliminary findings.

## Figures and Tables

**Table 1:** Baseline clinical characteristics of psoriasis patients.

Clinical Characteristic	N = 18
* General*	
Male gender, *n* (%)	11 (61.1)
Age (years)	49.1 ± 12.7
BMI (kg/m^2^)	27.3 ± 3.9
Current cigarette smoker, *n* (%)	12 (66.7)
* Disease characteristics*	
Age at disease onset	34.8 ± 13.0
Disease duration	14.3 ± 11.9
PASI at baseline	11.5 ± 11.7
* Difficult-to-treat locations*	
Genital	7 (38.9)
Scalp	6 (33.3)
Nails	5 (27.6)
Palmar/plantar	7 (38.9)
* Biologic therapy, n, (%)*	
Biologic naïve	14 (77.8)
Previous biologic	4 (22.2)
* Comorbidities, n (%)*	
Hypertension	6 (33.3)
Obesity	6 (33.3)
Diabetes mellitus	3 (16.7)
Dyslipidemia	2 (11.1)
Psoriatic arthritis	2 (11.1)
Thyroid disorder	1 (5.6)
HBV+/HCV+	1 (5.6)
QuantiFERON-TB Gold+	1 (5.6)

BMI = body mass index, HBV/HCV = hepatitis B/C virus, PASI = psoriasis area severity index, TB = tuberculosis.

## Data Availability

Not applicable.
